# Molecular Basis of Inflammation in the Pathogenesis of Cardiomyopathies

**DOI:** 10.3390/ijms21186462

**Published:** 2020-09-04

**Authors:** Emanuele Monda, Giuseppe Palmiero, Marta Rubino, Federica Verrillo, Federica Amodio, Francesco Di Fraia, Roberta Pacileo, Fabio Fimiani, Augusto Esposito, Annapaola Cirillo, Adelaide Fusco, Elisabetta Moscarella, Giulia Frisso, Maria Giovanna Russo, Giuseppe Pacileo, Paolo Calabrò, Olga Scudiero, Martina Caiazza, Giuseppe Limongelli

**Affiliations:** 1 Department of Translational Medical Sciences, University of Campania “Luigi Vanvitelli”, 80131 Naples, Italy; emanuelemonda@me.com (E.M.); g.palmiero@hotmail.it (G.P.); rubinomarta@libero.it (M.R.); fedeverrillo@gmail.com (F.V.); amodio.federica@yahoo.it (F.A.); fyrent@libero.it (F.D.F.); pacileoroberta@gmail.com (R.P.); fimianifabio@hotmail.it (F.F.); augustoesposito1990@gmail.com (A.E.); cirilloannapaola@gmail.com (A.C.); adelaidefusco@hotmail.it (A.F.); elisabetta.moscarella@gmail.com (E.M.); mariagiovanna.russo@unicampania.it (M.G.R.); gpacielo58@gmail.com (G.P.); paolo.calabro@unicampania.it (P.C.); martina.caiazza@yahoo.it (M.C.); 2 Dipartimento di Medicina Molecolaree Biotecnologie Mediche, University of Naples “Federico II”, 80138 Naples, Italy; gfrisso@unina.it (G.F.); olga.scudiero@unina.it (O.S.); 3 CEINGE-Biotecnologie Avanzate Scarl, Via G. Salvatore 486, 80138 Naples, Italy; 4 Task Force sugli Studi del Microbioma, University of Naples “Federico II”, 80138 Naples, Italy; 5 Institute of Cardiovascular Sciences, University College of London and St. Bartholomew’s Hospital, London WC1E 6DD, UK

**Keywords:** inflammation, cardiomyopathies, pathogenesis

## Abstract

Cardiomyopathies (CMPs) represent a diverse group of heart muscle diseases, grouped into specific morphological and functional phenotypes. CMPs are associated with mutations in sarcomeric and non-sarcomeric genes, with several suspected epigenetic and environmental mechanisms involved in determining penetrance and expressivity. The understanding of the underlying molecular mechanisms of myocardial diseases is fundamental to achieving a proper management and treatment of these disorders. Among these, inflammation seems to play an important role in the pathogenesis of CMPs. The aim of the present study is to review the current knowledge on the role of inflammation and the immune system activation in the pathogenesis of CMPs and to identify potential molecular targets for a tailored anti-inflammatory treatment.

## 1. Introduction

Cardiomyopathies (CMPs) represent a diverse group of heart muscle diseases, defined as myocardial disorders in which the heart muscle is structurally and functionally abnormal in the absence of hypertension, valvular heart disease, congenital heart disease and coronary artery disease sufficient to explain the observed myocardial abnormality. According to the recent classification of the working group on myocardial and pericardial diseases of the European Society of Cardiology (ESC) [1], they are grouped into specific morphological and functional phenotypes, and each phenotype is then subclassified into familial (genetic) and nonfamilial (nongenetic) forms. Familial CMPs are largely associated with sarcomeric and nonsarcomeric mutations. These conditions show extreme heterogeneity in phenotype expression and disease penetrance, limiting the hypothesis that genetic mutations alone may explain all clinical and pathophysiological features. Thus, CMP patients can present different clinical courses, ranging from an asymptomatic status with no adverse events and a similar longevity when compared to the general population, to a severe symptomatic phenotype that can present with heart failure, life-threatening arrhythmias, stroke or sudden cardiac death [2].

The development and progression of a cardiomyopathic phenotype depends on a complex interaction between several cellular signaling pathways, environmental stressors and individual genotypes. Our understating regarding the development and the progression of each cardiomyopathy has improved over the last decades. Among the several mechanisms implicated, inflammation seems to have an important role in the pathogenesis of CMPs [3,4,5].

Myocardial inflammation has recently emerged as a pathophysiologic process that contributes to cardiac hypertrophy, fibrosis and dysfunction in the context of heart disease [6]. Elevated inflammatory biomarkers represent a hallmark features of both heart failure with a preserved ejection fraction and heart failure with a reduced ejection fraction. The “cytokine hypothesis” has postulated that heart failure progresses, at least in part, as a result of the deleterious effects of the endogenous cytokine cascades on the myocardium and the peripheral circulation [7].

Moreover, the evidence that CMPs, such as heart failure, can be associated with elevated circulating levels of proinflammatory cytokines has provided a new research area that has revealed a potential role of inflammation and immune system activation in the pathogenesis of these disorders.

The aim of the present review is to summarize the current knowledge on the role of inflammation and immune system activation in the pathogenesis of CMPs and to identify potential molecular targets for a tailored anti-inflammatory treatment.

## 2. Cardiac Inflammation and Fibrosis: “The Vicious Cycle”

To explain their role in the pathogenesis of CMPs, we briefly describe the most important cellular and molecular mechanisms that contribute to cardiac inflammation and fibrosis. Inflammation and fibrosis occur in nearly all forms of myocardial injury and seem to play an important role in the pathogenesis of different myocardial diseases [8]. Myocardial injury may result as a consequence of environmental injury (such as myocardial ischemia or hemodynamic overload) or invading pathogens, and inflammation represents a physiological mechanism of defense against several different injuries, with the aim to eliminate the harmful stimuli. The inappropriate inflammatory response leads to the persistence of the harmful mechanism and results in chronic inflammation and myocardial fibrosis [9]. Fibrosis is a component of tissue repair that follows injury and is often associated with inflammation. While its objective is to preserve the tissue architecture, progressive fibrosis represents a pathologic condition and results in dysfunction, scarring and myocardial injury [10].

Several cell types play an important role in cardiac inflammation. First, neutrophils and monocytes migrate to the site of cardiac injury and release harmful mediators such as reactive oxygen species (ROS) and several proteases, in order to eliminate the factors that caused the cardiac injury. However, their response is nonspecific and could result in additional cardiac insult [11]. Thus, macrophages exposed to inflammatory signals assume a proinflammatory phenotype and sustain cardiac inflammation and injury-releasing inflammatory cytokines, and they stimulate fibroblasts and cardiomyocytes to adopt a proinflammatory phenotype [12]. Moreover, lymphocytes are involved in sustaining inflammation through several effectors [13]. Cardiomyocytes also secrete proinflammatory cytokines after myocardial injury. In particular, the secretion of IL6 and related cytokines is involved in cardiomyocytes’ hypertrophy and apoptosis; moreover, they favor the chronicity of inflammation and, subsequently, the macrophages’ infiltrations. Cardiac fibroblasts exposed to inflammatory cytokines (e.g., TNFα) develop an inflammatory phenotype and begin to secrete inflammatory cytokines such as IL1 and IL6, perpetuating the preexisting inflammation [14].

Several inflammatory pathways are involved in cardiac inflammation, such as the TNF-NF-κβ pathway (implicated in cardiac infection and injury) [15] or caspase-1 inflammasome pathways (activated during oxidative and cellular stress) [10]. Macrophages play a dominant role in the inflammatory pathways. They express various pattern recognition receptors, such as Toll-Like Receptors (TLRs).

**Figure 1:**
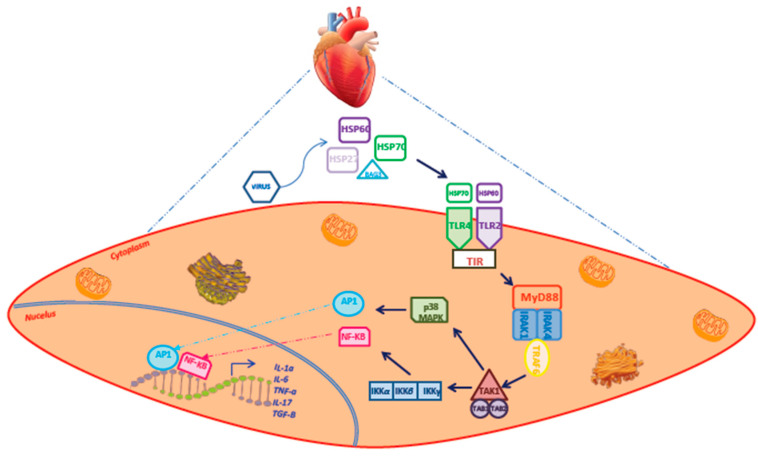
Schematic representation of NF-κB and MAPK pathways. Chaperone proteins are released by cardiac cells and bind to surface receptors on cardiac cell types activating specific signaling chronic inflammation. Cell surface toll-like receptors (TLR) are activated by Pathogen-Associated Molecular Patterns (PAMPs) or Danger-Associated Molecular Patterns (DAMPs) to initiate intracellular signaling. Myeloid Differentiation Primary Response 88 (MYD88) binds to Interleukin-1 Receptor-Associated Kinase 4 (IRAK4) and IRAK1/2. The IRAK complex interacts with TNF Receptor Associated Factor 6 (TRAF6). TRAF6 forms a complex with Mitogen-Activated Protein Kinase Kinase Kinase 7 (MAP3K7 or TAK1), MAP3K7 Binding Protein 1 (TAB1) and 2 (TAB2). TAK1 then activates the IκB Kinase (IKK) complex, thus activating the Nuclear Factor Kappa B (NF-κB). The released NF-κB translocates into the nucleus and mediates the expression of a number of proinflammatory cytokine genes. In addition, TAK1 can activate the Mitogen-Activated Protein Kinase (MAPK) signaling pathway. The MAPK signaling pathway can activate the transcription factor Activator Protein 1 (AP-1). The activation of NF-κB and AP-1 contributes to the expression of proinflammatory cytokines, such as IL-1, IL-6 and TNF-α.

TLRs are activated by molecular motifs from pathogens (i.e., Pathogen-Associated Molecular Patterns–PAMPs) or from endogenous material released by stressed or damaged cells (i.e., Microbe-Associated Molecular Patterns–MAMPs) [16]. The engagement of TLRs initiates downstream signaling cascades that regulate the expression of genes encoding proinflammatory cytokines and chemokines, in particular through the activation of the NF-κB and MAPK pathways [16] (Figure 1). Thus, the local inflammatory response is triggered by the ligation of TLRs, which give rise to ROS production and cytokine release.

The persistent activation of cardiac inflammatory pathways may lead to significant fibrotic changes, which represent a substrate for pathological myocardial remodeling. Myocardial fibrosis results in electromechanical disturbances and reduces the nutrient supply toward the myocardium, perpetuating a vicious cycle of fibrosis, myocyte death and inflammation [17].

## 3. Inflammation Mechanisms in Specific Cardiomyopathies

### 3.1. Arrhythmogenic Cardiomyopathy

Arrhythmogenic cardiomyopathy (ACM) is a heart muscle disease characterized by a fibro-fatty replacement of the right ventricular and/or left ventricular myocardium, which leads to anatomical, contractility and electrical rhythm abnormalities with life-threatening complications [18,19]. The predominant cause of the dominant-right ACM (ARVC) is represented by mutations in genes that encode components of desmosomes, the adhesive junctions that connect cardiomyocytes, and it leads to the loss of efficient electrical coupling with a consequent increased arrhythmic burden [20]. Apart from regulating a strong cell-cell adhesion, desmosomes have a key role in mediating signal transduction pathways. In particular, plakoglobin is involved in the regulation of transcription by competing with β -catenin in the Wnt/β -catenin signaling pathway through T cell/lymphoid–enhancing binding transcription factors [21]. The disruption of the desmosomes and adherens junctions leads to increased mechanical stress on the cardiomyocytes. This stress causes the activation of the Hippo pathway, an evolutionarily conserved pathway that controls the organ size by regulating cell proliferation, apoptosis and stem cell self-renewal [22]. This results in the inhibition of gene targets and promotes a proapoptotic and adipogenic phenotype. The dysregulation of calcium handling in the cardiomyocytes is thought to contribute to arrhythmogenesis [23]. The abnormal shuttling and tethering of both the ion channel and gap junction components have been suspected to be involved in arrhythmogenesis.

A tailored pharmacological treatment that directly targets the underlying disease mechanism in the various forms of ARVC is under investigation. Recently, an inhibitor of GSK3𝛽, a major regulator of Wnt/𝛽-catenin signaling identified in a zebrafish model of ARVC [24], showed a significant reduction in arrhythmias, myocardial damage and exercise-induced injury in mouse models of ARVC [24,25]. However, the chronic long-term use of Wnt agonists may have significant adverse events (including the increased risk of developing cancer), thus limiting the role of this pharmacological option as a treatment for ARVC patients.

On the other hand, dominant-left ACM (ALVC) is principally associated with nondesmosomal arrhythmia-associated variants (e.g., lamin A/C, phospholamban, filamin-C) [26,27,28] and usually manifests with left ventricular dilation, dysfunction and arrhythmias, falling into a “grey zone” in which a differential diagnosis with arrhythmogenic DCM is often difficult [29].

**Figure 2:**
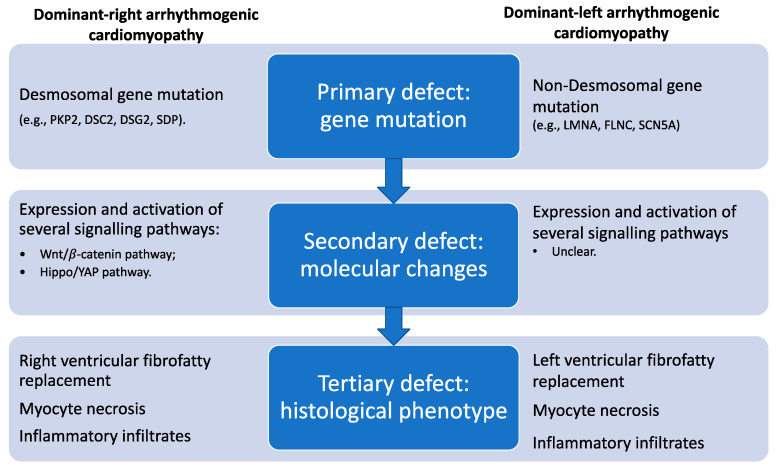
Schematic representation of the pathogenesis of arrhythmogenic cardiomyopathy. See text for details.

In ACM, the replacement of the myocardium by fibro-fatty tissue has been related to three principal mechanisms (Figure 2): apoptosis or programmed cell death, myocardial dystrophy, which may reflect a genetically determined atrophy, and inflammatory heart disease with a different clinical presentation ranging from acute myocarditis to diffuse fibrotic replacement, which in its severe form can involve both the right and the left ventricles and may lead to refractory biventricular heart failure that mimics DCM.

However, the pathophysiology of ACM is still partially unknown, and in recent years a relationship between myocardial inflammation and ACM has been described; in particular, the role of infectious agents has been proposed because of the common findings of patchy cell death with inflammatory infiltration in the myocardium [3], suggesting that infection of the myocardium may result in the activation of inflammatory mediators and adipose deposition, resulting finally in the disruption of cardiac adherens junctions, cardiomyocytes’ apoptosis and fibro-fatty replacement [30]. Recently, Campuzano et al. suggested the hypothesis that a genetically vulnerable myocardium may be predisposed to myocarditis [31]; in particular, they proposed that a genetic alteration in the desmosome may create the environment needed for the seeding of an infectious agent, which may subsequently lead to an inflammatory process.

Myocardial inflammation in ARVC can be analyzed though invasive procedures such as endomyocardial biopsies or through postmortem autopsy studies, and it was noted in the form of lymphocyte infiltrates in nearly two thirds of ARVC cases [3].

Moreover, myocardial inflammation in ARVC can also be identified noninvasively with the combined analysis of the plasma levels of inflammatory cytokines and cardiac scintigraphy [32]. In particular, compared to controls, ACM patients showed higher plasma levels of the inflammatory cytokines IL-1β, IL-6 and TNF-α and a higher myocardial ^67^Ga uptake [32]. There is evidence that these inflammatory cytokines can modulate the expression and function of ion channels, both by directly acting on cardiomyocytes and/or by inducing a systemic effect [33]. These changes may be involved in disease progression, heart failure development and ventricular arrhythmias. Recently, it has been shown that inflammatory mediators may play a role in the pathogenesis of ARVC, even in the absence of infiltrating inflammatory cells in the heart [34]. In particular, Asimaki et al. showed that a brief exposure to a low concentration of IL-17, IL-6 and TNF-α cytokines (implicated in granulomatous inflammation) caused the translocation of plakoglobin from cell-cell junctions to intracellular sites in cultured neonatal rat ventricular myocytes, and observed the myocardial expression of IL-17 and TNFα, as well as elevated serum levels of inflammatory mediators including IL-6R, IL-8, MCP1 and MIP1β in ARVC patients, supporting the role of the inflammatory mediators in the pathogenesis of ARVC [34]. Finally, the association of ARVC with the inflammatory response is expressed both by high CRP plasma levels and a high incidence of TV, even in the absence of morpho-functional anomalies of the right ventricle [35].

Similarly, inflammatory cell infiltrates have been described in patients with ALVC [36]. The subepicardial distribution of the lesions is similar to that reported in myocarditis [37]; in particular, CMR in ALVC often demonstrates patterns of subepicardial scarring with a predilection for the left ventricular inferior and lateral walls, similar to that seen following myocarditis [38].

Smith et al. [39] evaluated the clinical and genetic parameters of 107 patients with pathogenic desmoplakin (DSP) mutations and 81 patients with pathogenic plakophilin 2 (PKP2) mutations as a comparison cohort. The authors showed that episodes of acute myocardial injury occurred in 15% of patients with DSP and were strongly associated with LV late gadolinium enhancement. In four DSP cases with 18F-fluorodeoxyglucose positron emission tomography scans, acute LV myocardial injury was associated with myocardial inflammation that was initially misdiagnosed as cardiac sarcoidosis or myocarditis. Indeed, DSP mutations cause a unique form of ACM characterized by episodic left ventricular myocardial inflammation, fibrosis and systolic dysfunction, and DSP cardiomyopathy should be considered in the differential diagnosis for myocarditis and sarcoidosis. Moreover, the presence of any left ventricular systolic dysfunction in DSP cardiomyopathy indicates a substantial risk for severe ventricular arrhythmias, particularly when associated with ventricular ectopy and LGE.

In conclusion, these findings suggest that desmosomal dysfunction may stimulate the inflammatory response, thus favoring the recruitment of the inflammatory cells and further weakening the junctional stability. However, it is unclear if myocardial inflammation represents a primary insult to disease mutations or a secondary response to cardiomyocyte death.

### 3.2. Hypertrophic Cardiomyopathy

Hypertrophic cardiomyopathy (HCM) is a heart muscle disease characterized by left ventricular hypertrophy that is not solely explained by abnormal loading conditions (i.e., hypertension, valvular heart disease, congenital heart disease) [40,41]. In nearly half of cases, the disease is inherited as an autosomal dominant genetic trait caused by mutations in genes encoding sarcomeric proteins [42,43,44,45,46] and is characterized by an extremely interindividual variability in the phenotypic expression.

**Figure 3:**
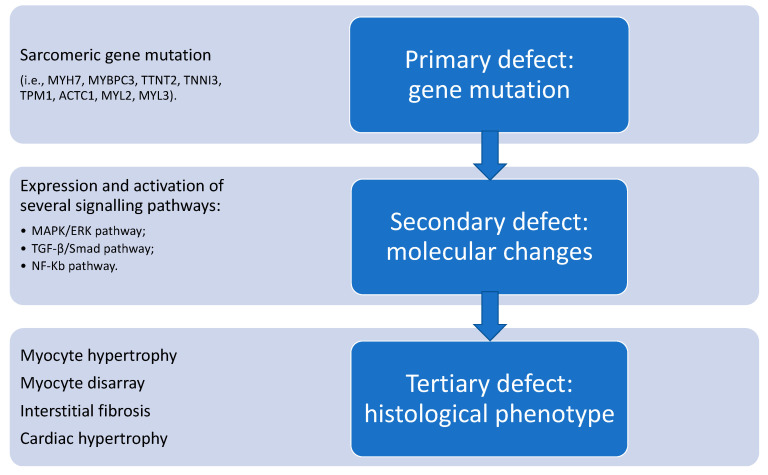
Schematic representation of the pathogenesis of hypertrophic cardiomyopathy. See text for details.

Different mechanisms are implicated in the pathogenesis of HCM (Figure 3). The primary determinant of the cardiac phenotype is represented by the causal gene mutation, which is directly responsible for several changes, such as an altered transcription rate and translation efficiency, as well as changes in the structure and function of the affected sarcomeric protein [47].

The initial defect causes a cascade of secondary molecular events, including the activation of the calcium-sensitive and stress-responsive molecular pathways that collectively mediate the programming of cardiac hypertrophy and induce the morphological and histological phenotypes that are recognized as HCM, principally represented by myocardial hypertrophy, myocyte disarray and myocardial fibrosis. The variability of the cardiac phenotype suggests that several epigenetic and environmental factors are involved in the pathogenesis of this disorder, but the mechanism by which the genetic mutation translates into the phenotype remains poorly understood [48]. Since the focus of HCM has typically been on myocardial hypertrophy, myocyte disarray and myocardial fibrosis, the role of inflammation and immune processes has been poorly characterized; however, it seems to have a pivotal role in the pathogenesis of HCM.

Several studies documented the leucocytes’ infiltration in the myocardium and the elevation of inflammatory cytokines in HCM patients [4,49,50], and the latter may play an important role in the status of HCM and its progression to the dilated-phase (hypokinetic end-stage) HCM. In particular, Zen et al. [49] evaluated the plasma levels of soluble Fas (sFas) or Fas ligand (sFas-L), TNF-α, and IL-6 in patients with idiopathic nonobstructive HCM and dilated-phase HCM and compared their levels with those present in normal subjects. They found that, in HCM patients, TNF-α and IL-6 were slightly higher when compared to normal subjects and sFas increased significantly. Moreover, sFas, TNF-α and IL-6 in dilated-phase HCM were significantly increased, and only IL-6 was significantly different when compared with nondilated HCM. In nondilated HCM, TNF-α was negatively correlated with fractional shortening, and in dilated-phase HCM patients high sFas was significantly associated with higher cumulative incidences of worsening heart failure. Thus, the proinflammatory cytokines might have significant detrimental effects on HCM patients.

Kuusisto et al. [51] evaluated the role of inflammation in the phenotypic expression of myocardial fibrosis in sarcomeric HCM patients. They observed that endomyocardial samples in patients with HCM showed myocyte hypertrophy, myofiber disarray, fibrosis (as expected), inflammatory cell infiltration and nuclear factor kappa B (NF-κB) activation. High-sensitivity CRP and inflammatory interleukins (i.e., IL-1β, IL-1RA, IL-6, IL-10) levels were significantly higher in HCM patients when compared with healthy controls. Moreover, the authors found a significant association between the degree of myocardial inflammatory cell infiltration and fibrosis in histopathological samples and late gadolinium enhancement (LGE) in cardiac magnetic resonance (CMR), suggesting that the inflammatory response was associated with myocardial fibrosis and that myocardial fibrosis in HCM was an active process modified by an inflammatory response. They suggested that a primary injury induces NF-κB upregulation, which is responsible for the inflammatory cytokine production, myocardial inflammatory infiltration and activation of fibroblasts, leading to myocardial fibrosis [51]. Since myocardial fibrosis is a predictor of ventricular arrhythmias and an end-stage hypokinetic state in HCM patients, the idea that myocardial fibrosis is a potentially modifiable process opens interesting clinical implications for preventing such complications.

Subsequently, Fang et al. [52] showed that several circulating inflammatory cytokines and peripheral inflammatory cells were associated not only with myocardial fibrosis, but also with the degree of hypertrophy and diastolic dysfunction, suggesting the potential role of the markers of systemic inflammation as biomarkers for the disease severity in HCM patients. Moreover, it has been shown that TNF-α and IL-6 cytokines may play an important role in HCM pathogenesis. In particular, it has been shown that TNF-α cardiac overexpression causes LV hypertrophy and premature death [53] and that IL-6 is a mediator of LV hypertrophy, myocardial fibrosis and LV dysfunction in response to pressure overload [54].

The trigger for early inflammation in HCM may be linked to cardiomyocyte disarray and sarcomere injury, mitochondrial oxidative stress, and microvascular disease with tissue injury; however, to date this trigger has not been clearly determined.

A recent study by Becker et al. explained the key role of neutrophil extracellular traps (NETs) in the pathogenesis of hypertrophic cardiomyopathy [55]. In response to a strong stimulation, leukocytes may release their nuclear contents (mainly consisting of DNA, chromatin, histones, elastase and cathepsin G), and these molecules physically trap cells (such as erythrocytes and platelets), fibrinogen and a variety of proteins involved in inflammatory response, fibrin deposition, platelet aggregation, endothelial cell injury and the activation of the intrinsic pathway of coagulation [56]. In particular, platelets activated by NETs release polyphosphates, which are prothrombotic and cause cardiac injury. Mitochondrial DNA also represents a trigger for inflammation after its internalization by cardiomyocytes, upregulating proinflammatory signaling pathways [57].

In brief, the stages of the pathogenesis of HCM can include a genetic predisposition, represented by a sarcomeric gene mutation in about 40–60% of patients, an early phase characterized by tissue level inflammation and NETs, an intermediate phase with platelet activation, microvascular thrombosis and transforming growth factor (TGF) β1 signaling, and a late phase of myocardial fibrosis, ventricular disarray and hypertrophy, finally responsible for the clinical features and adverse events. The animal HCM models provide an opportunity to determine if tissue level inflammation occurs, the underlying mechanisms that may involve ETs, and whether they participate in the phenotypic expression of HCM and in the transition from a hypertrophic state, characterized by myocyte hypertrophy to a dilated (hypokinetic end-stage) state with diffuse myocardial fibrosis and an impaired systolic performance.

### 3.3. Dilated Cardiomyopathy

The term dilated cardiomyopathy (DCM) is used to refer to a spectrum of heterogeneous myocardial disorders that are characterized by ventricular dilation and depressed myocardial performance in the absence of hypertension, valvular heart disease, congenital heart disease and ischemic heart disease [1]. This condition is not considered as a single disease entity, but rather as a nonspecific phenotype, which may be interpreted as the final common response of the myocardium to a number of genetic and environmental insults. The etiology of DCM can be classified within genetic and nongenetic causes, but there are circumstances in which a genetic predisposition may interact with environmental factors. In particular, genetic mutations account for 35% of DCM cases, and the majority of genetic DCM is inherited in an autosomal dominant pattern with a variable expressivity and penetrance [1,58]. DCM is genetically heterogeneous, and mutations in genes encoding cytoskeletal, sarcomeric, mitochondrial, desmosomal, nuclear membrane and RNA-binding proteins have all been linked to DCM. Thus, the pathological mechanisms that lead to DCM are diverse.

Next to the genetic cause, various acquired causes (infectious myocarditis, exposure to toxins/drugs and metabolic/endocrine disturbance) are responsible for the remaining portion of DCM patients [1,59].

The term dilated inflammatory cardiomyopathy (DCMi) refers to a broad group of disorders presenting with a dilated phenotype in which inflammation of the heart is the cause of myocardial dysfunction and can be viewed in contradistinction to a secondary immune response to other mechanisms of injury (i.e., ischemia or genetic CMP) [60]. Nevertheless, the presence of myocardial inflammation may be evidenced in most patients with advanced heart failure, regardless of the pathogenesis; thus, we have focused on the pathogenesis of DCM in which the inflammation represents the primary pathological event.

Infectious myocarditis is the leading cause of DCMi worldwide. The diagnosis of viral DCMi has been facilitated by the introduction of new-generation protein chain reaction (PCR) techniques in endomyocardial biopsy (EMB). The prevalence of specific pathogens involved in infectious myocarditis varies according to the geographical location, different molecular methods used and epidemiologic shift [61]. Noninfectious DCMi is usually associated with autoimmune disorders (i.e., sarcoidosis), eosinophilic cardiomyopathy (i.e., hypersensitivity myocarditis) and toxins/drugs exposure (i.e., cocaine) [62]. EMB studies have given evidence of a link between the initiation and progression of myocarditis to DMCi [59]: up to 30% of patients with myocarditis may develop DMC [63], and up to 50% of DMC cases show evidence of inflammation in the myocardium [64].

**Figure 4:**
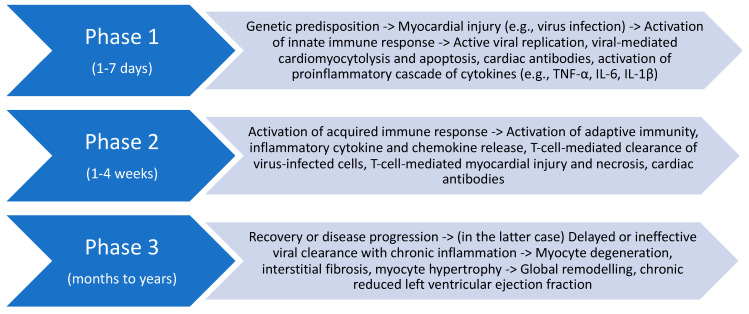
Schematic representation of the pathogenesis of dilated inflammatory cardiomyopathy. See text for details.

A pathophysiological model of DMCi development has been validated for some forms of infectious myocarditis and noninfectious pathogens. In this model, a myocardial injury occurs and triggers a chronological sequence comprised of three pathogenic phases (Figure 4) [62,65,66]:

(i) the myocardial injury is determined by a specific pathogen, directly and by nonspecific immunity. Then, the innate immune response, through recognition receptors such as TLR, determines a proinflammatory cascade of cytokines (IL-6 and IL-1β interleukins, TNF-α) and interferons when it detects pathogen-associated (infectious triggers) or damage-associated (noninfectious triggers) molecular patterns.

(ii) In the second phase, one to four weeks after the onset of the myocardial injury, there is a specific immune response with autoimmune or hypersensitivity features, based on the cross-reactivity of viral epitopes and some cardiac structures (molecular mimicry phenomenon), as well as the exposure of intracellular structures to the immune system, consequent T and B lymphocytes activation, and antibodies production against myocyte structures (myosin, troponin, beta-adrenoreceptors).

(iii) Finally, after several weeks to months, the complex interplay between the infectious or noninfectious myocardial damage and immune response may lead to ventricular dilatation and dysfunction.

Immunosuppressive therapy has been considered to be a promising approach to DCMi. However, large trials showed mixed results [67,68]. It has been shown that ongoing viral replication has negative prognostic implications and that virus-positive patients are less likely to respond to immunosuppression when compared to those without viral replication [69]. The lack of response to immunosuppressive therapy could be explained by the absence of a viral genome detection in early trials. Then, the current consensus is that myocarditis patients with biopsy-proven viral clearance by PCR may benefit from immunosuppressive agents [67]. However, large randomized trials are still underway.

## 4. Future Inflammation-Based Therapy in CMPs

The growing evidence for the major role of inflammatory mechanisms in CMPs has prompted the development of new targeted therapies in this setting. Although potential targets in HCM and in ACM have not yet been identified, novel targeted inflammatory treatments seem to be feasible in DCM.

Since the inflammatory cascade in infectious DCMi is triggered by a viral internalization by the common cellular receptor and by consequent intracellular signaling, its prevention could alter viral infection. Tyrosin kinase p56lck seems a logical target for pharmaceutical agents [70]. After the viral infection and the consequent immune response, many mechanisms have been established that could represent targets for therapy: antiviral treatment, plasmapheresis, use of hyperimmune globulin and immunosuppressive therapy are all possible strategies that are employable in patients with proven immune activation, but their efficacy should be documented. New strategies, relying on anticytokines, T-cell receptor vaccines and myosin-induced tolerance are in preclinical stages and offer promising results.

Additionally, the IL-1β blockade seems to be a promising novel target therapy. Recently, the anakinra (an IL-1β blockade) showed a beneficial effect on inflammation, myocardial performance and clinical status in acute decompensated heart failure (HF) patients [71]. Its safety and efficacy have been tested in a larger cohort of decompensated HF patients, showing no improvement in cardiac function or prognosis in terms of death or HF hospitalization at 24 weeks as compared to a placebo [72], probably because of being underpowered in terms of the outcome purpose. A randomized controlled phase 2B trial using anakinra versus standard care is underway (NCT03018834). Moreover, in the CANTOS trial [73], a double-blind, randomized, placebo-controlled trial that included 10,061 patients with myocardial infarction and inflammatory atherosclerosis denoted by high-sensitivity C-reactive protein (hsCRP) levels ≥2 mg/L, Canakinumab (a monoclonal antibody against IL-1β) showed a significant dose-dependent reduction in HF hospitalization and mortality in the HF subgroup when compared to a placebo.

The immunoadsorption and subsequent administration of polyclonal IgG has been proposed for chronic inflammatory autoimmunity with promising results [74]. In DCM patients with a high viral load, intravenous immunoglobulin seems beneficial [75].

## 5. Conclusions

Inflammation plays a central role in the disease pathogenesis and progression of myocardial disease. To date, there is no evidence to support the use of anti-inflammatory treatment in these patients, and the modulation of the inflammation remains a promising target for the treatment of CMPs. Additional studies are needed to further delineate the mechanisms and to identify novel target molecules.
